# Exploring the relationship between cathepsin and age-related macular degeneration using Mendelian randomization

**DOI:** 10.3389/fmed.2024.1460779

**Published:** 2024-11-06

**Authors:** Qiuyuan Wang, Shanjun Cai

**Affiliations:** ^1^Guizhou Branch of the Affiliated Hospital of Zunyi Medical University, National Clinical Research Center of the Eye Hospital of Guizhou Province, Key Laboratory of Eye Disease Characteristics of Guizhou Province, Zunyi, China; ^2^Department of Clinical Medicine, The First Clinical College, Zunyi Medical University, Zunyi, China

**Keywords:** cathepsins, age-related macular degeneration, Mendelian randomization, causal analysis, multivariable Mendelian randomization

## Abstract

**Purpose:**

Age-related macular degeneration (AMD) is the leading cause of low vision and even blindness in the elderly population worldwide. However, no studies have been conducted to analyze the causal relationship between the cathepsin family and AMD. The present study aimed to explore and analyze this potential association using Mendelian randomization (MR).

**Methods:**

In this study, AMD was classified into two types: exudative AMD and atrophic AMD. Inverse-variance weighting (IVW) was used as the main analysis method. The association between nine cathepsins and the two classifications of AMD were analyzed using multivariable Mendelian randomization (MVMR). Sensitivity analysis included Cochran’s Q-test and the MR-Egger intercept test.

**Results:**

Two-sample MR analysis showed that higher levels of cathepsin L2 were associated with a delay in the development of atrophic AMD (IVW: *p* = 0.017; OR = 0.885; 95% CI = 0.799–0.979). Reverse MR analysis indicated that cathepsin E levels were increased in individuals with atrophic (IVW: *p* = 0.023; OR = 1.058; 95% CI = 1.007–1.111) and exudative AMD (IVW: *p* = 0.018; OR = 1.061; 95% CI 1 = 1.010–1.115). MVMR analysis indicated a causal relationship between cathepsin G (IVW: *p* = 0.025; OR = 1.124; 95% CI = 1.014–1.245), cathepsin O (IVW: *p* = 0.043, OR = 1.158, 95% CI = 1.004–1.336), and exudative AMD after coordinating for other types of cathepsin.

**Conclusion:**

This study demonstrated a potential link between the cathepsin family and the onset of AMD. Elevated serum concentrations of cathepsin L2 may serve as a protective factor for atrophic AMD, while increased levels of serum cathepsin G and O concentrations may promote the development of exudative AMD. Besides, the development of AMD may be associated with elevated serum concentrations of cathepsin E.

## 1 Introduction

Age-related macular degeneration (AMD) is the leading cause of low vision and blindness in the elderly population (1). Currently, the global prevalence of AMD is approximately 8.69% (age range: 45–85 years), with the number of affected patients projected to increase to 288 million by 2040 (2). Therefore, preventing AMD risk factors has become an important area of research in clinical practice. AMD-related risk factors include age, immune system-related genetic variants, smoking, obesity, excessive cholesterol intake, and various known cardiovascular metabolic factors (3). In addition, some studies have shown that hyperglycemia can affect the development of AMD through the accumulation of highly stable advanced glycation end products, oxidative stress, and hemodynamic perturbations related to inflammatory responses, such as mitochondrial dysfunction (4). Cathepsins represent a group of lysosomal proteolytic enzymes that play an important role in maintaining cellular homeostasis (5). Common cathepsins belong to the papain superfamily of cysteine proteases (6). They are integral to almost all physiological and pathophysiological cellular processes, such as protein and lipid metabolism, autophagy, antigen presentation, growth factor receptor recycling, cellular stress signaling, extracellular matrix degradation, and lysosome-mediated cell death (7). The cathepsin family is closely involved in regulating proinflammatory signaling pathways, and cathepsin D and S can promote the degradation of the photoreceptor outside the retina (8). Moreover, Thomas (9) found a causal relationship between cathepsin F and early AMD.

However, no study has explored the mechanism further. Some scholars believe that the abnormal regulation of cathepsin activity may be related to the occurrence and development of AMD. However, no previous study in China has been conducted to analyze systematically whether a potential link exists between cathepsins and the occurrence and development of AMD (2). An increasing number of studies have revealed the role of genetics in disease etiology with the advancement of genomics. Mendelian randomization (MR) relies on genome-wide association studies (GWAS) using one or more genetic variants as instrumental variables (IVs) for causal analysis. These variables are strongly associated with the exposure and are unaffected by confounding factors. MR studies can infer the causal effect of exposure on outcomes (10). In this context, this study analyzed the potential causal associations between different types of tissue proteins and both AMD classifications using two-sample and multivariate MR methods.

## 2 Materials and methods

### 2.1 Instrumental variables (IVs)

The IV for tissue proteins (μg/L) was obtained from the INTERVAL study, which included 3,301 Europeans (11). The data source is https://questions.mrcieu.ac.uk. The cathepsin-related IV screening conditions for MR analysis are as follows: *p* < 5×10^−8^, linkage disequilibrium (LD, *r*^2^ ≤ 0.001), satisfying Hardy–Weinberg balance, and a genetic distance of <10,000 kb (12).

### 2.2 Outcome data source

Based on clinical presentation, the Age-related Eye Disease Study team classified AMD into AMD-free, early, middle, and advanced stages. Two distinct manifestations emerged in the late stages of AMD (13). One is the development of confluent areas of atrophy involving photoreceptors and retinal pigment epithelium, known as geographic atrophy (atrophic AMD). The other is the growth of abnormal blood vessels in the macular region, referred to as neovascular AMD (exudative AMD) (13). Thus, the AMD data were obtained from the FinnGen database. This study divided the outcome (AMD) into previous studies of exudative AMD (4,848 case group, 252,277 controls; European population) and atrophic AMD (6,065 case group, 251,042 controls; European population). Table 1 shows the details.

**Table 1:** Two-sample forward MR analysis.

Database	Data name	The first author	Sample capacity	The year of publication	Race	Sex	Website
IEU	Cathepsins	Jialin Li	3301	2023	European	Men and women	https://gwas.mrcieu.ac.uk
FinnGen	Dry age-related macular degeneration	NA	257107	2023	European	Men and women	https://storage.googleapis.com/finngen-public-data-r9/summary_stats/finngen_R9_DRY_AMD.gz
	Wet age-related macular degeneration	NA	257125	2023	European	Men and women	https://storage.googleapis.com/finngen-public-data-r9/summary_stats/finngen_R9_WET_AMD.gz/

## 3 Analytical methods

**Figure 1:**
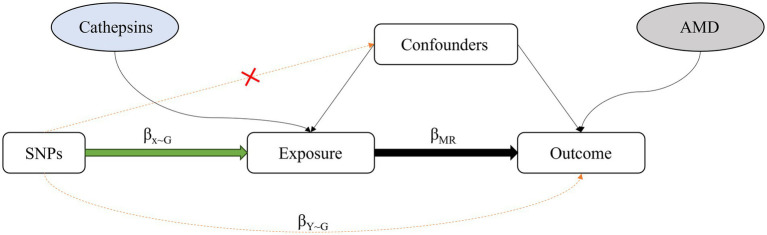
Diagram of the approach used by Mendelian randomization studies, which compare the observed genotype-outcome association with the expected genotype-outcome association: β_x ~ G_: regression coefficient of the genetic variant-exposure association. β_Y ~ G_: regression coefficient of the genetic variant-outcome association. β_MR_: regression coefficient of the exposure-outcome association.

MR refers to an analytic approach to assess the causality of an observed association between a modifiable exposure or risk factor and a clinically relevant outcome (14). It uses genetic variation as an IV to analyze whether exposure would have a causal effect on the outcome (14). For two-sample MR, the causal effect of the exposure (X is cathepsins) on the outcome (Y is AMD) via the GIV (G) can then be estimated by β_MR_ = β_Y ~ G_/β_x ~ G_; β_MR_ (known as the Wald ratio estimate) represents the causal effect estimate obtained from β_Y ~ G_ and β_x ~ G,_ the regression coefficients obtained from the regression of the outcome on the GIV and the regression of the exposure on the GIV, respectively (15) (Figure 1). MVMR is an extension of MR that allows for the causal effects of multiple exposures on an outcome to be estimated (16).

**Figure 2:**
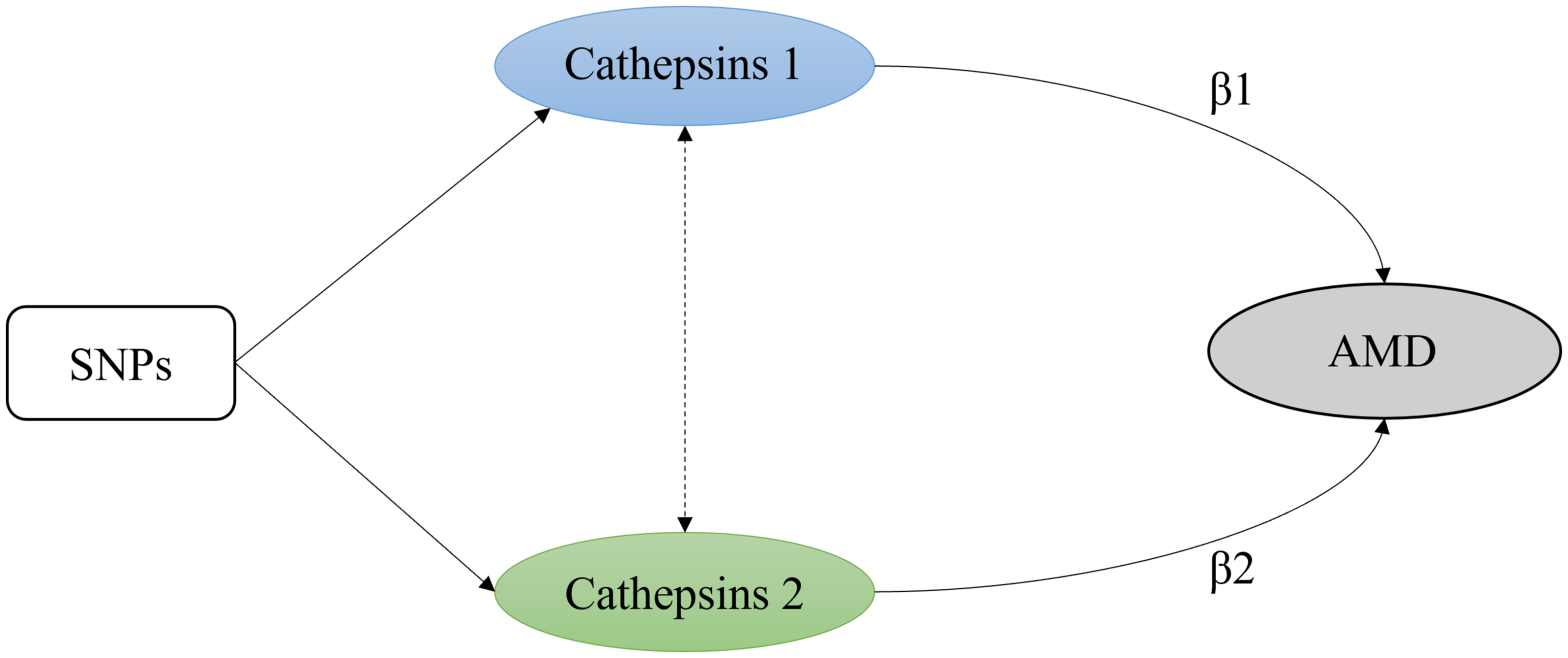
A simple multivariable Mendelian randomization model with two exposures. β1 and β2 are the intercept and effects of exposure on the outcome.

MVMR estimates the “direct” causal effects of each exposure included in the estimation of the outcome, conditional on the other exposures included in the model (17). It is particularly useful where two or more potentially related exposures are of interest, and the researcher wishes to understand whether both exposures exert a causal effect on the outcome or, as described later, where one exposure is potentially a mediator of another exposure. With individual-level data, MVMR is implemented through two-stage least-squares regression of the model: Y = β_0_ + β_1_X_1_ + β_2_X_2_ + V_y,_ where Y is the outcome of interest; X_1_ and X_2_ are the exposures of interest; β0, β1, and β2 are the intercept and effects of X_1_ and X_2_ on the outcome, respectively. V_y_ is a random error term that is assumed to be normally distributed (18) (Figure 2).

The selected IVs must meet three criteria. First, they should be highly correlated with the exposure. Second, the SNP should not confound the relationship between exposure and outcome. Finally, the SNP cannot be associated with the outcome through any pathway other than exposure. When the last two conditions are violated, the SNP is considered to exhibit horizontal pleiotropy (19).

In previous studies, inverse variance weighting (IVW) has been used as the primary method for estimating the overall effect size (20). In particular, the causal effect estimates from each genetic variant are combined using an IVW meta-analysis framework. Thus, the IVW method is a weighted average of the causal effects derived from the genetic variants. This approach is akin to fitting a weighted linear regression of the associations between the instruments and the outcome, with the intercept term set to zero. Notably, this method assumes that all instruments are valid and that no pleiotropic effects exist, meaning the genetic variants are not associated with multiple exposures. Thus, any differences in the causal estimates derived from each genetic variant can be attributed to sampling variability, adhering to the homogeneity assumption (21, 22). Supplementary methods such as MR-Egger (23) and weighted median (WM) (24) were used to verify the robustness of the MR results.

In short, in the presence of pleiotropy, one could fit a weighted linear regression of the associations between the instruments and the outcome while assuming an unconstrained intercept term (in contrast to the IVW approach, where the intercept term is constrained and set to zero), resulting in the MR-Egger regression method (20). The slope of the MR-Egger regression is a robust estimate of the causal effect accounting for potential horizontal pleiotropy. An estimator of the WM method (15) is a median, where the individual MR estimates are weighted proportionally according to their precision. When up to 50% of genetic variants are invalid instruments, a causal effect can be estimated as the median of the weighted ratio estimates using the reciprocal of the variance of the ratio estimate as weights (24). MR analysis (including IVW, MR-Egger, and WM) was performed using the R TwoSampleMR package.

Sensitivity analysis and statistical tests were performed in this study to evaluate the validity of the hypotheses. The heterogeneity of SNPs was judged using Cochran’s Q test, with a *p*-value of >0.05, indicating a lack of heterogeneity (12). The MR-PRESSO global test and the MR-Egger intercept were used to identify outliers and horizontal pleiotropic effects (25).

The MR-Egger intercept represents the average multidirectional effect (intercept *p* < 0.05), and the slope can produce robust multidirectional MR estimates.

The MR-PRESSO outliers test was used to correct for pleiotropy by removing or lowering outliers when pleiotropy was significant (tested here using *p* < 0.05) (25).

Multivariate MR is an extension of the two-sample MR (19). This study not only included the causal relationship between individual cathepsins and AMD but also explored the association between nine cathepsins and AMD through multivariate MR. In addition, this study included two AMD classifications as exposure factors by using cathepsins as the outcome to assess reverse causality and demonstrate the existence of bidirectional causality. These reverse MR analyses used the same GWAS dataset described above.

## 4 Results

### 4.1 Two-sample MR analysis clarified the causal relationship between nine cathepsins and different subtypes of AMD

**Figure 3:**
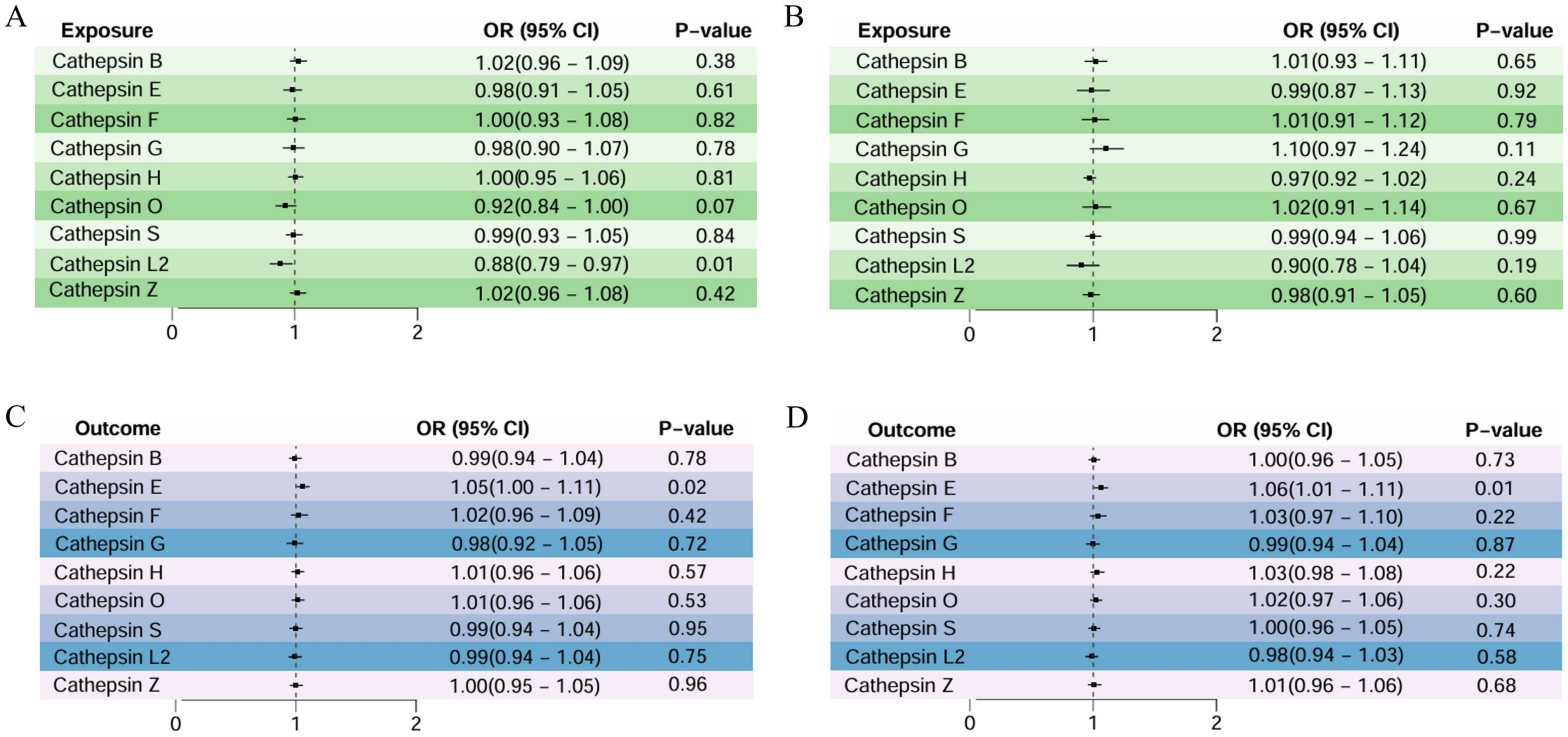
Two-sample MR analysis: **(A)** forward analysis of atrophic AMD, **(B)** forward analysis of exudative AMD, **(C)** reverse analysis of atrophic AMD, and **(D)** reverse analysis of exudative AMD.

As detailed in Figure 3, a two-sample MR analysis of nine cathepsins (B, E, F, G, H, L2, O, S, and Z) and both AMD classifications was performed to assess the effect of nine cathepsins on AMD subtypes. The results showed that for atrophic AMD, the increased concentrations of serum cathepsin L2 delayed its progression (IVW: *p* = 0.017; OR = 0.885; 95% CI = 0.799–0.979).

However, a weak association existed between cathepsin O and atrophic AMD, with elevated serum cathepsin O concentrations protecting against it (WM: *p* = 0.038; OR = 0.882; 95% CI = 0.783–0.993). For exudative AMD, none of the nine cathepsins showed a significant correlation with them. None of the above studies showed pleiotropy or heterogeneity. All the analyses are detailed in Table 2.

**Table 2:** Two-sample forward MR analysis.

Cathepsin	SNPs	Inverse variance weighted		MR-Egger		Weighted median	
		OR (95%CI)	*p*	OR (95%CI)	*p*	OR (95%CI)	*p*
Cathepsin B							
Atrophic AMD	18	1.02 (0.96–1.09)	0.38	1.01 (0.87–1.17)	0.85	0.97 (0.89–1.07)	0.65
Exudative AMD	18	1.01 (0.93–1.11)	0.65	1.07 (0.87–1.31)	0.48	1.03 (0.93–1.14)	0.45
Cathepsin E							
Atrophic AMD	9	0.98 (0.91–1.05)	0.61	0.99 (0.88–1.12)	0.95	0.96 (0.86–1.06)	0.46
Exudative AMD	9	0.99 (0.87–1.13)	0.92	0.90 (0.72–1.12)	0.39	0.94 (0.84–1.05)	0.28
Cathepsin F							
Atrophic AMD	10	1.00 (0.93–1.08)	0.82	1.03 (0.86–1.24)	0.73	1.00 (0.91–1.10)	0.91
Exudative AMD	10	1.01 (0.91–1.12)	0.79	1.19 (0.91–1.55)	0.22	0.96 (0.86–1.07)	0.48
Cathepsin G							
Atrophic AMD	11	0.98 (0.90–1.07)	0.78	1.05 (0.87–1.26)	0.57	0.99 (0.88–1.12)	0.97
Exudative AMD	11	1.10 (0.97–1.24)	0.11	1.18 (0.90–1.54)	0.24	1.12 (0.97–1.28)	0.10
Cathepsin H							
Atrophic AMD	10	1.00 (0.95–1.06)	0.81	0.97 (0.90–1.04)	0.47	0.99 (0.94–1.04)	0.77
Exudative AMD	10	0.97 (0.92–1.01)	0.24	0.92 (0.86–0.98)	0.04	0.95 (0.90–1.00)	0.07
Cathepsin L2							
Atrophic AMD	11	0.88 (0.79–0.97)	0.01	0.77 (0.59–1.01)	0.09	0.93 (0.81–1.07)	0.34
Exudative AMD	11	0.90 (0.78–1.04)	0.19	1.39 (1.03–1.88)	0.05	0.92 (0.79–1.08)	0.34
Cathepsin O							
Atrophic AMD	12	0.92 (0.84–1.00)	0.07	1.04 (0.85–1.28)	0.64	0.88 (0.78–0.99)	0.03
Exudative AMD	12	1.02 (0.91–1.14)	0.67	1.02 (0.78–1.33)	0.86	0.99 (0.87–1.13)	0.95
Cathepsin S							
Atrophic AMD	23	0.99 (0.93–1.05)	0.84	0.89 (0.80–0.98)	0.03	0.91 (0.84–0.99)	0.03
Exudative AMD	23	0.99 (0.94–1.06)	0.99	0.93 (0.84–1.03)	0.22	0.94 (0.86–1.03)	0.20
Cathepsin Z							
Atrophic AMD	13	1.02 (0.96–1.08)	0.42	0.95 (0.87–1.05)	0.38	0.98 (0.90–1.06)	0.66
Exudative AMD	13	0.98 (0.91–1.05)	0.60	0.96 (0.86–1.06)	0.49	0.96 (0.88–1.04)	0.37

AMD, age-related macular degeneration; CI, confidence interval; OR, odds ratio.

Reverse MR analysis was performed in this study to explore whether reverse causality existed. The occurrence of atrophic AMD caused increased cathepsin E (IVW: *p* = 0.023; OR = 1.058; 95% CI = 1.007–1.111), and the MR-Egger intercept and the MR-PRESSO analysis showed no significant pleiotropy (0.757 and 0.811, respectively). Exudative AMD also led to elevated cathepsin E (IVW: *p* = 0.018; OR = 1.061; 95% CI = 1.010–1.115).

Moreover, the MR-Egger intercept and the MR-PRESSO analysis showed that the results did not show significant pleiotropy (0.291 and 0.248, respectively). The rest of the analysis did not demonstrate a causal link between the two AMD classifications and other cathepsins (Table 2).

### 4.2 MVMR analysis clarified the causal relationship between nine cathepsins and different subtypes of AMD

**Figure 4:**
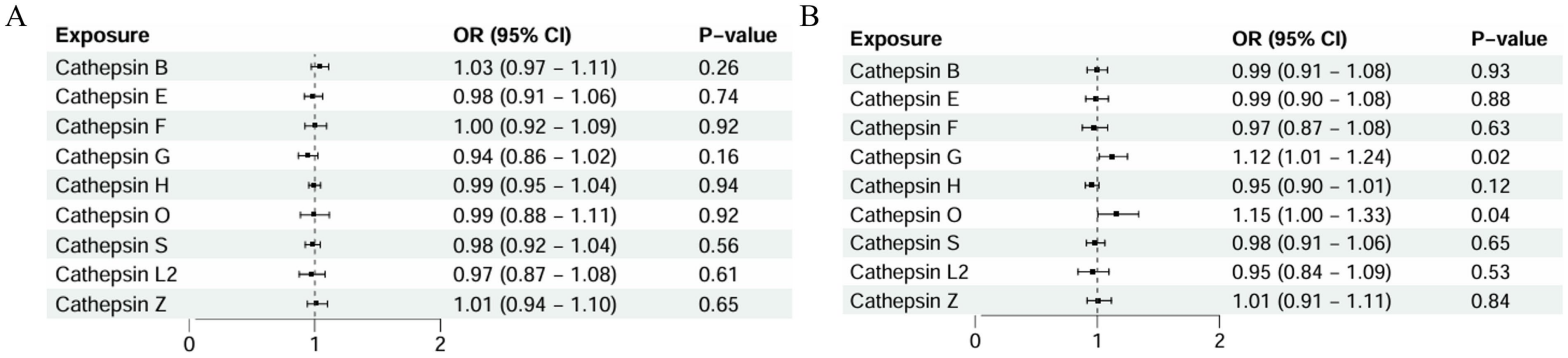
Multivariate MR analysis: **(A)** atrophic AMD analysis and **(B)** exudative AMD analysis.

In this study, the relationship between the genetic propensity of multiple cathepsins and different subtypes of AMD was assessed by multivariate MR. The results showed that after the coordination of other types of cathepsin enzymes, serum cathepsin G (IVW: *p* = 0.025; OR = 1.124; 95% CI = 1.014–1.245) and the increased concentration of cathepsin O (IVW: *p* = 0.043; OR = 1.158; 95% CI = 1.004–1.336) could promote the occurrence of exudative AMD. For atrophic AMD, no significant cathepsins were found after the coordination of other types of cathepsins (Figure 4 and Table 3). The above analysis results do not have pleiotropy and heterogeneity.

**Table 3:** Two-sample reverse MR analysis.

Cathepsin	SNPs	Inverse variance weighted		MR-Egger		Weighted median	
		OR (95%CI)	*p*	OR (95%CI)	*p*	OR (95%CI)	*p*
Cathepsin B							
Atrophic AMD	21	0.99 (0.94–1.04)	0.78	0.99 (0.91–1.07)	0.92	1.00 (0.93–1.08)	0.86
Exudative AMD	20	1.00 (0.96–1.05)	0.73	0.97 (0.90–1.04)	0.44	1.00 (0.94–1.07)	0.84
Cathepsin E							
Atrophic AMD	21	1.05 (1.00–1.11)	0.02	1.03 (0.95–1.12)	0.35	1.05 (0.98–1.11)	0.12
Exudative AMD	20	1.06 (1.01–1.11)	0.01	1.00 (0.93–1.08)	0.89	1.04 (0.98–1.10)	0.11
Cathepsin F							
Atrophic AMD	21	1.02 (0.96–1.09)	0.42	0.96 (0.87–1.07)	0.56	1.00 (0.93–1.08)	0.84
Exudative AMD	20	1.03 (0.97–1.10)	0.22	0.97 (0.88–1.07)	0.61	1.02 (0.95–1.08)	0.49
Cathepsin G							
Atrophic AMD	21	0.98 (0.92–1.05)	0.72	0.99 (0.89–1.11)	0.92	1.02 (0.95–1.09)	0.47
Exudative AMD	20	0.99 (0.94–1.04)	0.87	0.98 (0.90–1.07)	0.68	1.01 (0.96–1.07)	0.49
Cathepsin H							
Atrophic AMD	21	1.01 (0.96–1.06)	0.57	0.97 (0.89–1.05)	0.54	1.00 (0.93–1.08)	0.86
Exudative AMD	20	1.03 (0.98–1.08)	0.22	0.98 (0.90–1.06)	0.71	1.05 (0.98–1.12)	0.12
Cathepsin L2							
Atrophic AMD	21	0.99 (0.94–1.04)	0.75	0.96 (0.88–1.04)	0.39	0.98 (0.91–1.05)	0.61
Exudative AMD	20	0.98 (0.94–1.03)	0.58	0.99 (0.91–1.07)	0.82	1.00 (0.94–1.06)	0.91
Cathepsin O							
Atrophic AMD	21	1.01 (0.96–1.06)	0.53	0.98 (0.90–1.06)	0.71	1.01 (0.94–1.09)	0.64
Exudative AMD	20	1.02 (0.97–1.06)	0.30	1.00 (0.93–1.08)	0.81	1.03 (0.96–1.10)	0.31
Cathepsin S							
Atrophic AMD	21	0.99 (0.94–1.04)	0.95	1.04 (0.96–1.13)	0.30	0.99 (0.92–1.05)	0.76
Exudative AMD	20	1.00 (0.96–1.05)	0.74	1.02 (0.95–1.10)	0.44	1.01 (0.95–1.07)	0.68
Cathepsin Z							
Atrophic AMD	21	1.00 (0.95–1.05)	0.96	0.96 (0.88–1.04)	0.36	1.02 (0.94–1.09)	0.58
Exudative AMD	20	1.01 (0.96–1.06)	0.68	0.95 (0.88–1.03)	0.31	1.01 (0.95–1.07)	0.70

AMD, age-related macular degeneration; CI, confidence interval; OR, odds ratio.

## 5 Discussion

This study revealed a potential causal relationship between the cathepsin family and the occurrence of AMD through MR analysis, thereby providing a favorable reference for further studies.

AMD is the major cause of low vision and even blindness in the elderly population (1). The number of patients with AMD worldwide is expected to reach 288 million in 2040 (2). The prevalence of AMD in people older than 70 years in China is 20.2% (2). The number of patients with AMD continues to increase with the aging of China’s population. The authors of the previous studies believe that the onset of AMD, a multifactorial disease, is associated with many risk factors, such as age, immune system-related genetic variants, smoking, obesity, excessive cholesterol intake, and known cardiovascular and metabolic factors (3). In addition, a study showed that the development of AMD is closely associated with numerous activated microglia and macrophages (2). However, no study has proposed definite observations related to the onset of AMD. Given the high blindness rate of the disease, exploring and analyzing the surveillance indicators of AMD pathogenesis is valuable for early diagnosis and treatment.

Previous studies have found that oxidative stress may affect the occurrence and development of AMD through microglia. In normal retinal tissue, continuous monitoring of harmful stimuli is performed by microglia, which are mostly confined to the plexiform layer, where they exhibit complex branching processes to sense the local retinal microenvironment (26).

These cells play an important role in retinal homeostasis and contribute to neuroprotection against transient pathophysiological insults. However, when stress is permanent, persistent microglial inflammatory responses may cause changes in retinal integrity and induce neuronal death. These changes lead to retinal degeneration and may also be caused by direct damage to glial cells by stress (27). Active microglia phagocytose retinal myelin debris and promote retinal regeneration. However, the ability of microglia to maintain immune surveillance and tissue repair decreases with age (28). Microglial senescence is associated with the production and release of proinflammatory cytokines, which are involved in the pathogenesis of AMD and other retinal neurodegenerative diseases (29). In addition, under oxidative stress, with the occurrence of aging retinal pigment epithelium (RPE), the production and accumulation of advanced glycation end products (AGEs) and the activation of AGE receptors (RAGE) are enhanced (30). AGE receptors are present in cells, such as endothelial cells, pericytes, microglia, monocytes, and macrophages (30). Experimental studies have shown that exposure of RPE cells to the RAGE ligand AGEsorS100B can cause retinal tissue damage through RPE-mediated VEGF expression, leading to pathological angiogenesis (31). Therefore, abnormal microglial function plays an important role in the occurrence and development of AMD.

The cathepsin family is involved in protein and lipid metabolism, autophagy, and antigen presentation and has great value for cell homeostasis (7). In the absence of external stimuli, cathepsins are generally affected by transcription, translation, and epigenetic regulation, and extracellular cathepsins can accumulate in the extracellular environment by activating immune cells, osteoclasts, fibroblasts, glial cells, endothelial cells, and smooth muscle cells (32). Cathepsin release plays a role when pathological conditions, such as cancer, inflammation, and immune imbalance, occur (32). Previous studies showed that cathepsins are important in the activation of microglia during chronic neuroinflammation (33, 34). This finding proves that cathepsins promote the development of AMD by activating microglia in response to external stimulation. As one of the characteristics of exudative AMD, the production mechanism of retinal neovascularization is also the focus of the current research. Wang (34) found that cathepsins can effectively affect the expression of proteins closely related to angiogenesis, such as phosphorylated endothelial-type nitrogen oxide synthase and phospho-glycogen synthase kinase-3 protein. Moreover, cathepsins can promote angiogenesis in response to hypoxia and ischemic stress.

Moreover, Jan (35) found that dysregulation of cystatin (cathepsin inhibitors) in humans may increase susceptibility to exudative AMD. This finding also provides further evidence for the potential role of cathepsins in exudative AMD.

Based on previous studies, the present study used multiple MR methods to comprehensively analyze the potential causal relationship between the cathepsin family and atrophic and exudative AMD.

The findings suggest that cathepsin L 2 may be a protective factor for atrophic AMD, while cathepsin G and O are risk factors for exudative AMD. In addition, atrophic and exudative AMD may be accompanied by increased cathepsin E concentrations. Previous studies have found that the cathepsin E-sTRAIL axis is involved in communication between microglia and neurons during the progression of Alzheimer’s disease (36). Given that Alzheimer’s disease and AMD are diseases of aging, this indirectly suggests that cathepsin E may also play an important role in the pathogenesis of AMD and is closely associated with the cathepsin E-sTRAIL axis (36). When other types of cathepsins were adjusted in multivariate analysis, no causal relationship existed between cathepsins and atrophic AMD. This result may be due to the functional compensation of other cathepsins, and multivariate MR analysis helped mitigate these potential biases that may affect traditional observational studies (12). This study explored and analyzed the possible causal relationship between the cathepsin family and AMD pathogenesis through MR, thereby providing a reference for the subsequent exploration of the effective monitoring indicators of AMD pathogenesis. Given that the pathogenesis of AMD is mediated by multiple factors and the cathepsin family is involved in many cellular physiological processes, further research is needed to analyze the specific link between the two.

With the continuous development of medical technology and science, the early screening, diagnosis, and treatment of AMD have become the focus of ophthalmologists. The results of this study provide new monitoring indicators for the early screening of AMD.

This study demonstrated a potential link between the cathepsin family and AMD pathogenesis, where elevated serum cathepsin L 2 concentration may be a protective factor for atrophic AMD.

Moreover, elevated serum concentrations of cathepsin G and O may promote the development of exudative AMD. However, the development of AMD may be accompanied by elevated serum cathepsin E concentrations.

Given the high blindness rate of AMD, recognizing and controlling the risk factors for AMD are crucial for reducing its prevalence and enabling early diagnosis and treatment.

Although this MR-designed investigation has several strengths that complement traditional epidemiological studies, it also has some limitations to be considered. First, the study was limited to individuals of European ancestry, which suggests that our findings should not be directly extrapolated to other populations. Second, while we did not observe evidence of pleiotropy for the causal association using different MR approaches, there remains a possibility that the variants used in the MR confer a risk of AMD through a pleiotropic pathway. Therefore, further MR analysis using individual-level data should be conducted to evaluate the potential causal relationship between the cathepsin family and the risk of AMD. In addition, further studies, such as ablation experiments, should be conducted to elucidate the underlying mechanism, which will help verify these findings.

## Data Availability

The original contributions presented in the study are included in the article/supplementary material, further inquiries can be directed to the corresponding author.
